# *Cupriavidus pinatubonensis* AEO106 deals with copper-induced oxidative stress before engaging in biodegradation of the herbicide 4-chloro-2-methylphenoxyacetic acid

**DOI:** 10.1186/s12866-017-1119-y

**Published:** 2017-10-30

**Authors:** Nanna Bygvraa Svenningsen, Mette Damgaard, Maria Rasmussen, Danilo Pérez-Pantoja, Ole Nybroe, Mette Haubjerg Nicolaisen

**Affiliations:** 1 Section for Microbial Ecology and Biotechnology, Department of Plant and Environmental Sciences, University of Copenhagen, Thorvaldsensvej 40, Frederiksberg C, Denmark; 2 Programa Institucional de Fomento a la Investigación, Desarrollo e Innovación, Universidad Tecnológica Metropolitana, Ignacio Valdivieso 2409, San Joaquín, Santiago, Chile

**Keywords:** Oxidative stress, ROS, *ohr*/*osmC*, Copper, PA degradation, *Cupriavidus pinatubonensis*

## Abstract

**Background:**

Microbial degradation of phenoxy acid (PA) herbicides in agricultural soils is important to minimize herbicide leaching to groundwater reservoirs. Degradation may, however, be hampered by exposure of the degrader bacteria to toxic metals as copper (Cu) in the soil environment. Exposure to Cu leads to accumulation of intracellular reactive oxygen species (ROS) in some bacteria, but it is not known how Cu-derived ROS and an ensuing oxidative stress affect the degradation of PA herbicides. Based on the previously proposed paradigm that bacteria deal with environmental stress before they engage in biodegradation, we studied how the degradation of the PA herbicide 2-methyl-4-chlorophenoxyacetic acid (MCPA) by the model PA degrader *Cupriavidus pinatubonensis* AEO106 was affected by Cu exposure.

**Results:**

Exposure of *C. pinatubonensis* in batch culture to sublethal concentrations of Cu increased accumulation of ROS measured by the oxidant sensing probe 2,7-dichlorodihydrofluorescein diacetate and flow cytometry, and resulted in upregulation of a gene encoding a protein belong to the Ohr/OsmC protein family. The *ohr*/*osmC* gene was also highly induced by H_2_O_2_ exposure suggesting that it is involved in the oxidative stress response in *C. pinatubonensis*. The increased ROS accumulation and increased expression of the oxidative stress defense coincided with a delay in the catabolic performance, since both expression of the catabolic *tfdA* gene and MCPA mineralization were delayed compared to unexposed control cells.

**Conclusions:**

The current study suggests that Cu-induced ROS accumulation in *C. pinatubonensis* activates a stress response involving the product of the *ohr*/*osmC* gene. Further, the stress response is launched before induction of the catabolic *tfdA* gene and mineralization occurs.

## Background

Degradation of the phenoxy acid (PA) herbicides 2,4-dichlorophenoxyacetic acid (2,4-D) and 2-methyl-4-chlorophenoxyacetic acid (MCPA) by soil microorganisms is normally a rapid process, and in experimental soil systems the pesticides are completely degraded within 30 days [1]. Nevertheless, PA herbicides often persist in the soil environment under natural conditions leading to leaching to groundwater reservoirs. The herbicides reach the groundwater even though PA degraders are commonly found in natural soil environments [2]. This suggests that the full mineralization potential of degrader organisms is not as efficiently expressed under in situ soil conditions as seen in laboratory setups. One reason could be the harsh conditions encountered in a soil environment. In soil, the bacteria are confronted with suboptimal conditions caused by for instance fluctuations in substrate availability, pH, temperature, water availability, as well as exposure to toxic, anthropogenic compounds [3–5]. These environmental factors might impose stress that compromise physiological processes in the bacteria and thereby hamper the catabolic potential present in the microbial community.

Co-contamination with metals has previously been shown to reduce degradation of PAs during remediation of contaminated soil [6]. In agricultural soil, the metal copper (Cu) accumulates as Cu^2+^ ions or Cu-complexes, often as a consequence of the application of Cu-containing manure from pig productions, where Cu is extensively used as a growth promoter [7]. Although Cu is essential for many biological functions, it becomes a stressor and may even become toxic when available in higher concentrations [8, 9]. Several studies indicate that free Cu ion activities correlate well with observed toxicological effects, suggesting that free Cu^2+^ constitutes the main toxic species responsible for inhibitory effects towards soil microorganisms [10]. Understanding the link between Cu accumulation and impact on PA degradation in agricultural systems is central in order to protect groundwater resources.

Cu is a redox-active metal that may induce oxidative stress in bacteria [11, 12]. Excess of Cu leads to damage on cell components as lipids, proteins and DNA [13, 14]. One type of Cu damage is caused by a Fenton reaction leading to formation of highly reactive hydroxyl radicals [11]. Further, free Cu^+^ can destabilize iron-sulfur clusters in important dehydratase enzymes [15]. Oxidative stress arises as a result of increased cellular production of reactive oxygen species (ROS) e.g. hydrogen peroxide (H_2_O_2_), hydroxyl (OH·)- and superoxide (O_2_·^−^) radicals, followed by a subsequent intracellular accumulation of these to levels that exceed the defense capacity of the cell [16]. The involvement of ROS and oxidative stress in response to Cu accumulation in PA degrading strains has however not previously been investigated.

Bacteria have evolved numerous defense mechanisms to keep the intracellular ROS level low, including enzymatic scavenging by superoxide dismutases and catalases [17]. Launching a defense response nevertheless comes with a cost. As expression of the defense system uses elements of the same transcriptional machinery as the catabolic pathways, induction of the defense system may interfere with the expression of catabolic genes. Hence, even subinhibitory levels of ROS potentially impede biodegradation despite the presence of cognate pollutant substrates. For example, the toluene- and xylene-degrading model organism *Pseudomonas putida* mt-2 downregulates the catabolic *xyl* genes in response to oxidative- and other stress inducing conditions in pure culture [18, 19]. Based on these findings, the authors proposed the paradigm that this organism responds to stressful conditions by transferring its transcriptional machinery to adapt to a given stressor before it turns on its catabolic machinery for pollutant degradation [11]; hence, stress endurance prevails over degradation of potential carbon substrates. Whether this response is a general trait for degrader organisms is currently unknown, but it could be one of the explainations why biodegradation of pollutants does not always take place at the expected rates in the environment.

The model PA degrader *Cupriavidus pinatubonensis* JMP134 carries the full set of *tfd* genes encoding the enzymatic pathway for complete mineralization of the PA herbicides 2,4-D and MCPA, and is able to utilize these as sole carbon- and energy sources [20]. This bacterium, as well as the isogenic tetracyclin-resistant derivate *C. pinatubonensis* AEO106 [21], has been extensively studied in relation to understanding xenobiotic degradation. Indeed several studies on expression of the *tfdA* gene, i.e. the gene catalyzing the first step in the sequential degradation of MCPA, have revealed a close link between catabolic gene expression and active mineralization both in controlled pure cultures and in soil microcosms [1, 20, 22]. Yet, its functional performance in relation to specific environmental stressors, e.g. elevated Cu concentrations, has neither been investigated at the functional nor at the genetic level.

In the current study, we tested the hypotheses that 1) amendment with Cu^2+^ imposes ROS accumulation in *Cupriavidus pinatubonensis* and 2) *C. pinatubonensis* deals with stress release before engaging in catabolic processes. We worked with a polyphasic approach that evaluated the impact of Cu on intracellular ROS accumulation, viability, PA mineralization, and expression of both the catabolic *tfdA* gene as well as identified a putative oxidative stress response gene in *C. pinatubonensis* AEO106. The presented data support that the previously proposed paradigm on the trade-off between stress response and catabolic degradation of carbon for growth holds for a wider range of bacteria.

## Results

### Impact of H_2_O_2_-stress on *tfdA* expression, and establishment of the *ohr/osmC* gene as a genetic marker for oxidative stress conditions

**Fig. 1:**
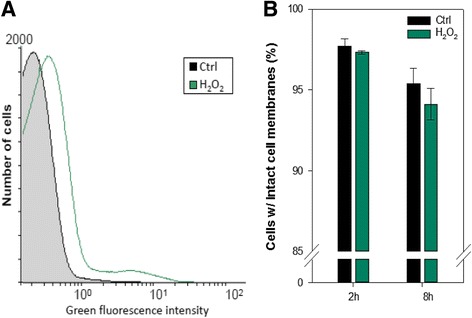
**a** Determination of intracellular ROS accumulation of cells exposed to 1 mM H_2_O_2_. The histogram shows ROS accumulation detected as green fluorescence from ROS-dependent oxidation of the ROS-sensitive probe H_2_DCF-DA following 30 min of incubation in the presence of H_2_O_2_. The experiment was repeated twice in triplicates; the histogram here shows fluorescence values from one representative replica. **b** Viability of cells following 2 and 8 h of exposure to 1 mM H_2_O_2_ measured as cells with intact membranes not stained by propidium iodide. Data are mean values from triplicate cultures from one representative experiment (the experiment was repeated twice). Error bars represents standard deviations

**Fig. 2:**
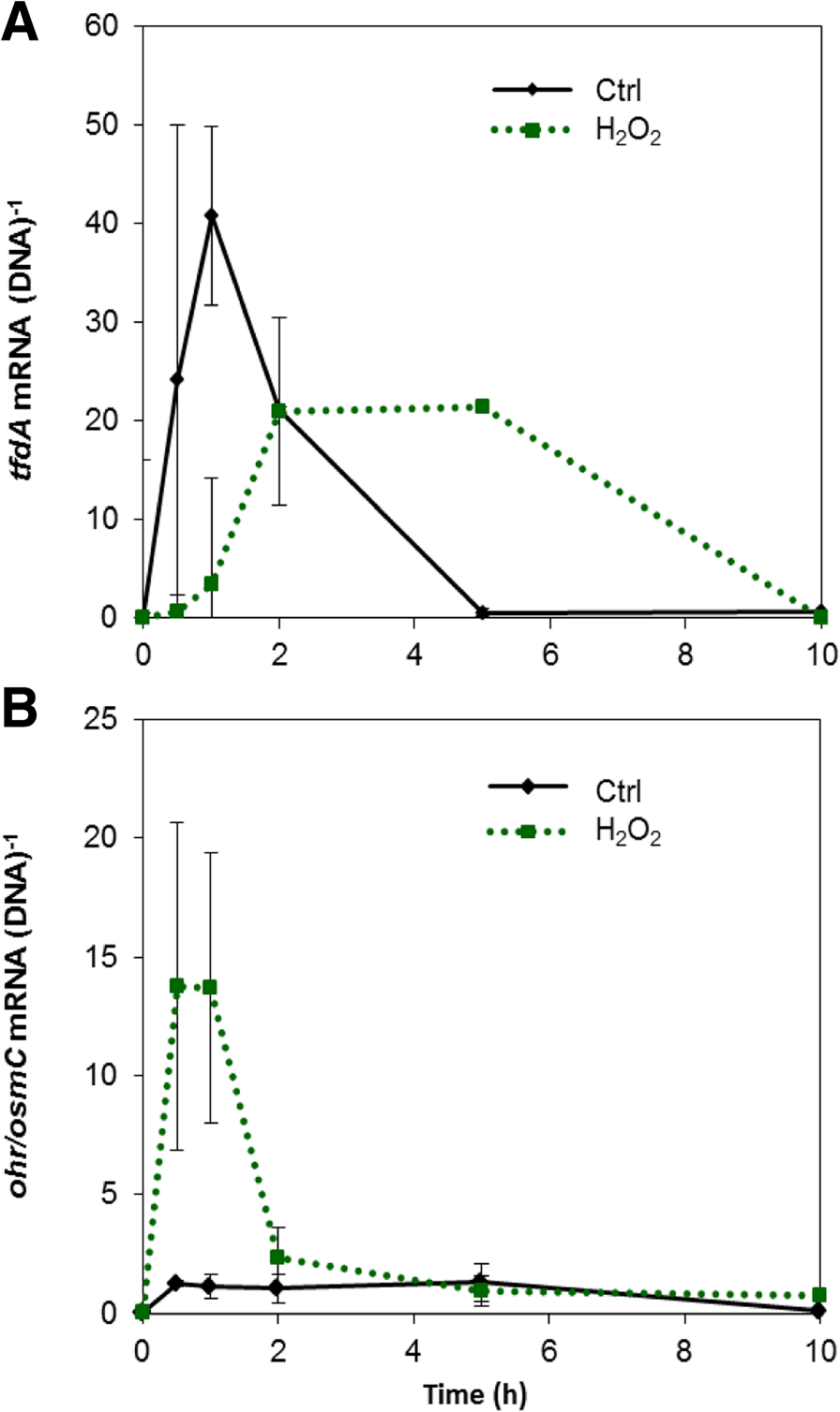
Gene expression by cells exposed to 0 (control) or 1 mM H_2_O_2_ measured by qPCR as mRNA normalized to the DNA copy number of the corresponding gene. **a** Expression of *tfdA* involved in the first step of MCPA degradation. **b** Expression of the *ohr/osmC*-like gene putatively involved in a response to oxidative stress in *C. pinatubonensis* AEO106. Data are mean values from triplicate cultures from one representative experiment (the experiment was repeated twice). Error bars represent standard errors of means

In initial pure culture experiments *C. pinatubonensis* was exposed simultaneously to MCPA and hydrogen peroxide (H_2_O_2_). H_2_O_2_ served as an external ROS source that rapidly enters cells by diffusion. For cells exposed to 1 mM H_2_O_2_ and 25 mg L^−1^ MCPA the average ROS-dependent green fluorescence intensity was 2.3-fold higher than for the control cells after 30 min as measured by the oxidant sensitive probe 2,7-dichlorodihydrofluorescein diacetate (H_2_DCF-DA) (Fig. 1a). This increase in intracellular ROS coincided with a delayed expression of the *tfdA* gene i.e. the gene catalyzing the first step in the sequential degradation of MCPA in *C. pinatubonensis* AEO106. For control cells, *tfdA* expression peaked 1 h after MCPA addition, whereas a broader peak was observed between 2 and 5 h in H_2_O_2_-treated cells (Fig. 2a). No significant differences in viability were detected between the control and the H_2_O_2_-treated cells within 8 h after the exposure (t-test, *p* > 0.05) (Fig. 1b) based on propidium iodide and SYBR Green staining. Hence, the delayed *tfdA* expression in response to H_2_O_2_ could be ascribed to a specific physiological response rather than cell death.

To investigate if the slower upregulation of *tfdA* in cells exposed to H_2_O_2_ corresponded with a rapid stress release, a putative oxidative stress responsive gene in *C. pinatubonensis* AEO106 had to be identified for comparison. As no such gene has previously been identified, we performed a search for genes in the *C. pinatubonensis* AEO106 genome encoding proteins homologous to proteins previously found to be upregulated in response to conditions inducing oxidative stress in *C. necator* H16 [23]. One of the proteins with the highest expression under the conditions tested by Schwartz and coworkers [23] is the organic hydroperoxide resistance protein Ohr. In *C. pinatubonensis* AEO16 the product of the gene with locus tag REUT_RS28250 shows 84% amino acid sequence identity to Ohr from H16, and is classified as an osmotically inducible protein, OsmC, by Pfam search. As Ohr/OsmC enzymes belong to a protein family, which is involved in the break-down of hydroperoxides [24, 25], REUT_RS28250 was tested for its response in H_2_O_2_-stressed cells. The gene is referred to as *ohr/osmC* hereafter, as no further attempt to reveal the specific identity of the gene was made in the current study.

Expression of *ohr/osmC* was induced after 30 min both in the control and the H_2_O_2_-treated cells. However, for H_2_O_2_-treated cells the up-regulation was approximately 10 fold higher than for control cells (Fig. 2b). The expression was constant throughout the time span of 8 h in the control cells, whereas H_2_O_2_-treated cells displayed a clear peak in expression between 30 min and 2 h, i.e. corresponding to the duration of the delay in *tfdA* expression. Thus, the *ohr/osmC* gene seemed to be involved in a response against H_2_O_2_-induced oxidative stress.

### Response to ROS induced by Cu exposure

Cells grown in minimal media amended with MCPA were exposed to three different concentrations of Cu (10, 30 and 50 μM) to examine whether Cu induces ROS accumulation and potential oxidative stress in *C. pinatubonensis* AEO106, and if so, whether Cu-induced oxidative stress hampers the degradation of MPCA.

**Fig. 3:**
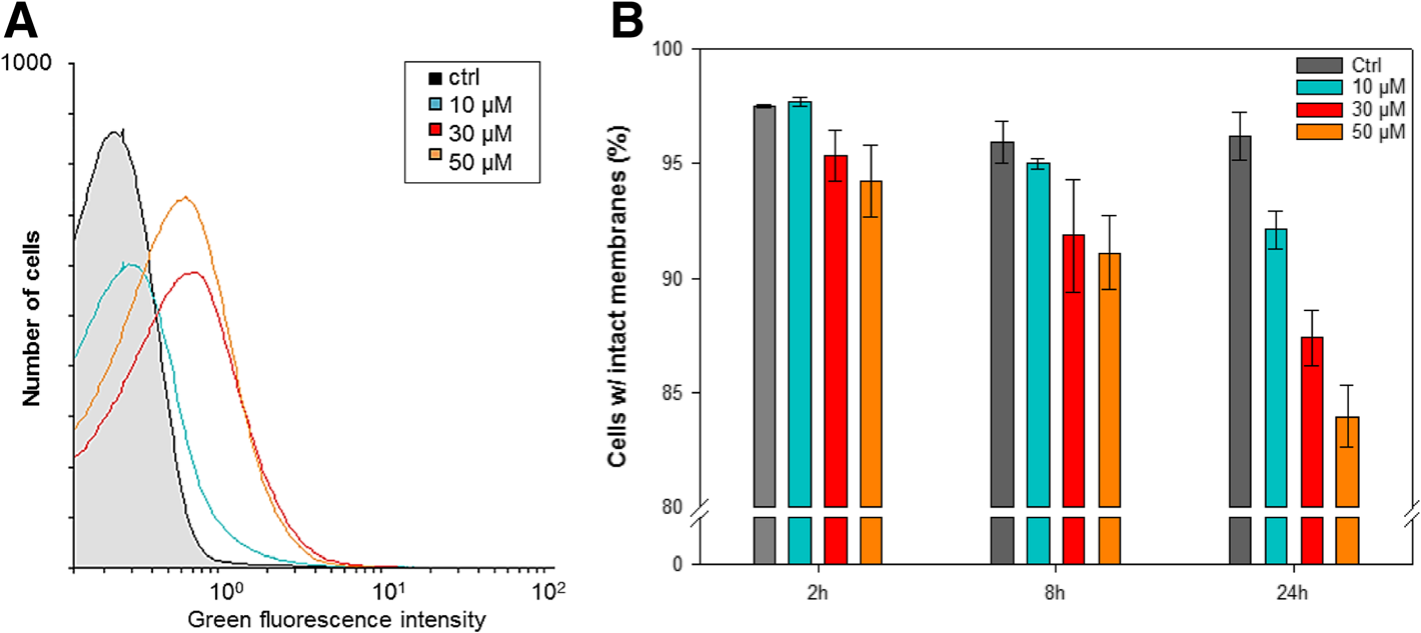
Determination of intracellular ROS accumulation and viability of cells exposed to 0–50 μM CuSO_4_. **a** Histogram showing ROS accumulation detected as green fluorescence from ROS-dependent oxidation of the ROS-sensitive probe H_2_DCF-DA following 30 min of incubation in the presence of Cu. The histogram shows data from a representative experiment. **b** Viability of cells following 2–24 h of exposure to CuSO_4_ measured as cells with intact membranes not stained by propidium iodide. Data are mean values from triplicate cultures from one representative experiment (the experiment was repeated twice). Error bars represents standard deviations

Figure 3a shows that a 30-min exposure to Cu led to an increase in ROS-dependent green fluorescence for all tested Cu concentrations. Longer exposure times did not lead to changes in ROS accumulation (data not shown). For the 10 μM Cu treatment the average fluorescence intensity was 13% higher than for the control cells, which is visualized by the thickened tail and a right-shifted population profile in the histogram. No differences were found between the 30 μM and 50 μM incubations where the average fluorescence intensity increased by 30% compared to the control cultures without Cu amendment. Exposure to 10 μM Cu did not cause a significant reduction in the amount of cells with intact cell membranes within the first 8 h (t-test, *p* > 0.05); however, over 24 h a significant (t-test, *p* < 0.05), but small decrease in cells with intact membranes were observed for the cells exposed to 10 μM Cu. The highest concentration of Cu (50 μM) caused significant cell death (t-test, p < 0.05) already after 8 h, although viable cells with intact cell membranes accounted for >80% of the total cells still after 24 h (Fig. 3b).

**Fig. 4:**
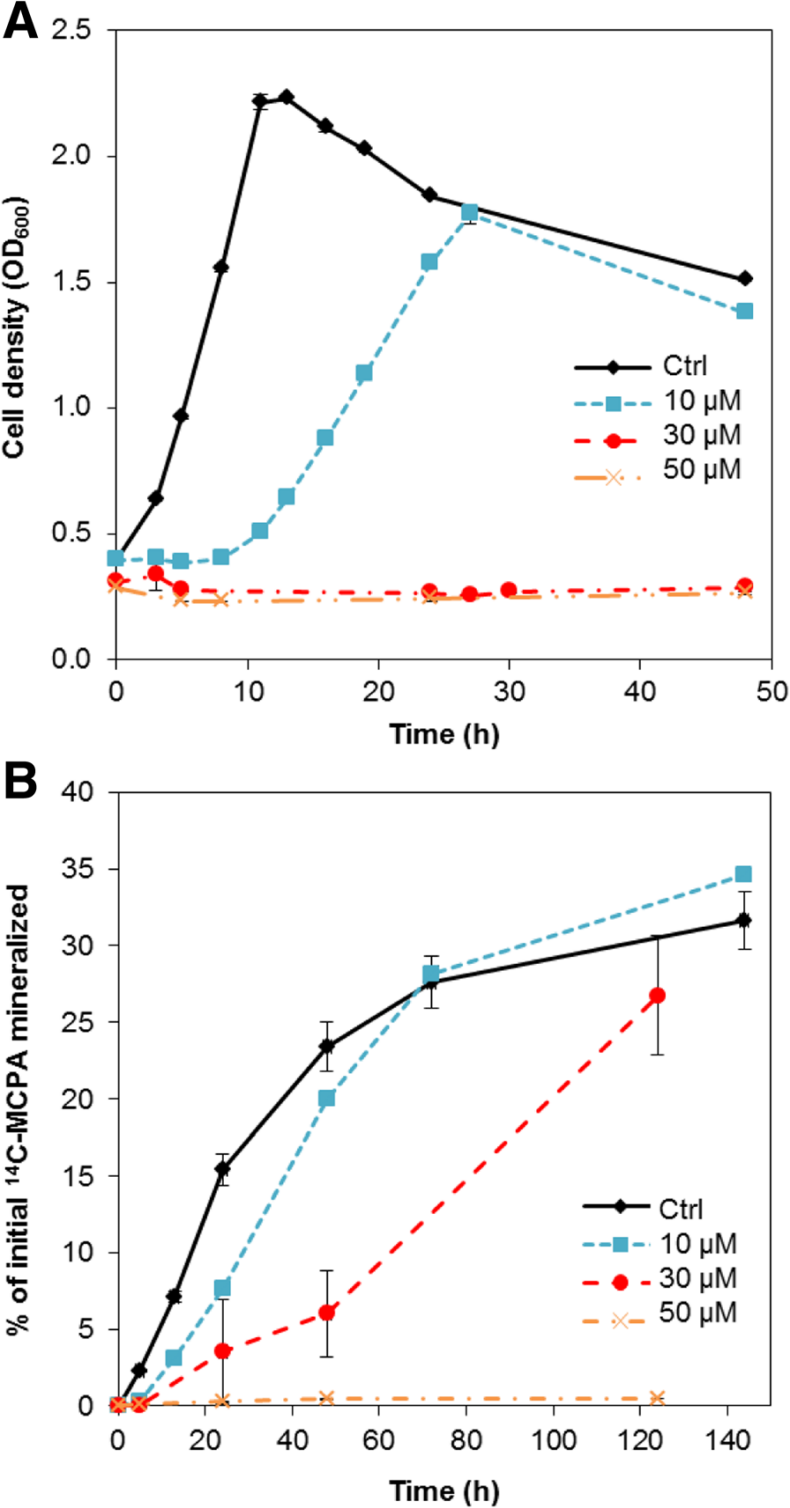
Growth and MCPA mineralization by *C. pinatubonensis* AEO106 under exposure to 0–50 μM CuSO_4_. **a** Growth measured as optical density at 600 nm. **b** Mineralization of ^14^C–MCPA measured as accumulation of ^14^C–CO_2_ compared to total amount of ^14^C–MCPA added to the cultures. Data are mean values from triplicate cultures from one representative experiment (the experiment was repeated twice). Error bars represents standard error of means

The increased amount of intracellular ROS was concurrent with delayed or even impaired growth. Figure 4a shows that non-treated cells started growing immediately, while cells exposed to 10 μM Cu started growing after a lag phase of between 8 and 11 h. Higher concentrations of Cu inhibited cell growth completely during the time course of the experiment.

Mineralization of MCPA was affected by Cu in a concentration-dependent manner. Cells exposed to 10 μM were slower at initiating mineralization than control cells, which corresponded to the delayed growth (compare Fig. 4a and b), but after 72 h the level of mineralization was similar to that observed for the control cells. Though cells exposed to 30 μM Cu did not grow, they were still able to mineralize MCPA after a longer lag phase.

**Fig. 5:**
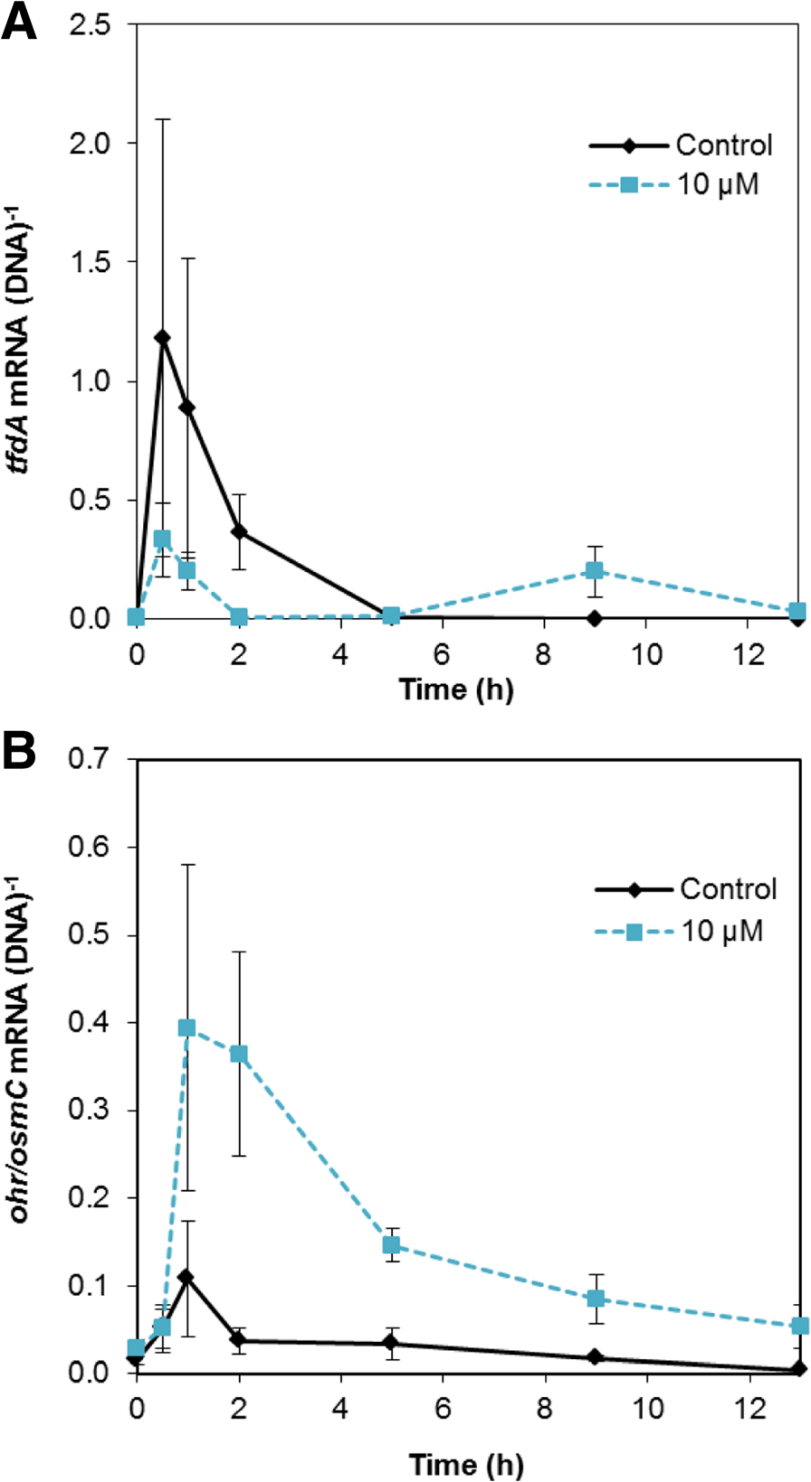
Gene expression by cells in liquid DMM + MCPA exposed to 0 (control) or 10 μM CuSO_4_ measured by RT-qPCR as mRNA normalized to the DNA copy number of the corresponding gene. **a** Expression of the catabolic *tfdA* gene. **b** Expression of the putatively oxidative stress responsive *ohr*/*osmC*-like gene. Data are mean values from triplicate cultures from one representative experiment (the experiment was repeated twice). Error bars represent standard error of means

Expression of *tfdA* was determined within the first 13 h for the control cells and for cells exposed to 10 μM Cu. Cells from both treatments upregulated *tfdA* within the first hour (Fig. 5a), but the expression level in the control cells was ~3.5 times higher than in the Cu-exposed cells. After 5 h *tfdA* expression was again downregulated in the control cells; in Cu-treated cells a downregulation appeared already after 1 h. Interestingly, a second *tfdA* expression peak in Cu-treated cells appeared after 9 h, matching with the onset of mineralization (Fig. 4b). Hence Cu delayed and decreased *tfdA* expression and only a second expression peak lead to efficient MCPA mineralization.

Subsequently, the expression of *ohr/osmC* was analyzed to examine if a putative ROS protective mechanism is in play under Cu stress, as observed for cells under H_2_O_2_ stress. The expression of *ohr/osmC* was upregulated ~7 times in control cells after 1 h, but the upregulation was transient and expression quickly went back to a low and steady level. In the Cu-exposed cells the upregulation was at least 30-fold and lasted for at least 5 h (Fig. 5b). After 5 h the expression level in the Cu exposed cells was not statistically significant different from the expression level in the control cells (t-test, *p* > 0.05).

Hence, exposure to a sublethal concentration (10 μM) of Cu did not prevent induction of gene expression in *C. pinatubonensis*. Rather, the reduced induction of *tfdA* coincided with a marked upregulation of *ohr/osmC*, a gene likely to be involved in an antioxidative response in this bacterium.

## Discussion

Little, if anything is known about if and how oxidative stress induced by physicochemical conditions that are relevant for agricultural soil influences degradation of PA herbicides. Oxidative stress hampers expression of catabolic genes in pure cultures of hydrocarbon-degrading pseudomonads [19]; hence, an oxidative stress scenario might influence pesticide-degrading bacteria and their ability to express catabolic genes in a comparable way. In the current work we therefore studied the oxidative stress response caused by Cu exposure at genetic and physiological levels in the model PA degrader strain *C. pinatubonensis* AEO106 in order to address the question whether stress is dealt with before biodegradation is initiated.

We exposed *C. pinatubonensis* in liquid culture to concentrations of Cu^2+^ from 10 to 50 μM. These concentrations are comparable to the concentrations of water extractable Cu that can be found in contaminated agricultural soils [26]. Further, these concentrations are considerably lower than those used to select for Cu-resistant bacteria from soil [26, 27].

Production of antioxidant enzymes e.g. catalases and superoxide dismutases (SODs) are among the major defense lines used by bacteria to exacerbate accumulation of intracellular ROS [8], and detection of their activity is frequently used as evidence for an oxidative stress response [23, 28, 29]. Nevertheless, since changes in gene expression (most often) is the first and most direct response to an environmental change, the expression of a wide array of oxidative stress responsive genes has been studied under various conditions [30–32]. For *C. pinatubonensis* AEO106/JMP134 no stress responsive genes have been described thus far. We previously identified a gene annotated as a catalase in these strains, but remarkably the gene did not show any response to H_2_O_2_ (unpublished data). Interestingly, we on the other hand determined a strong induction of an *ohr/osmC* gene in response to H_2_O_2_. Ohr (organic hydroperoxide resistance protein) and OsmC (osmotically inducible protein C) are homologous proteins belonging to a family of enzymes that are involved in breaking down organic hydroperoxides [33, 34]. Although the proteins belonging to the OsmC subfamily initially were recognized for their role in protection against osmotic stress [34], they are also reported to be involved in the defense against oxidative stress [25, 32, 35, 36]. A study by Saikolappan et al. [37] showed that *osmC* homologues in *Mycobacterium* sp. were upregulated as a response to temperature variation and H_2_O_2_ exposure, and recently Svenningsen et al. [32] detected increased expression of *osmC* in *Pseudomonas putida* exposed to H_2_O_2_. In *Mycoplasma genitalium*, which has the smallest genome among self-replicating bacteria, no genes encoding catalases or SODs have been found [38]. Instead Zhang and coworkers [38] have localized an *osmC* homologue, which is crucial in the oxidative stress defense of this bacterium. The conservation of *osmC* in a genome-streamlined bacterium suggests that the gene possesses an important biological function. In contrast to OsmC, the Ohr proteins seem to function specifically against organic hydroperoxides and not against inorganic hydroperoxides [24, 39].

In the current study we observed induction of the *ohr/osmC* gene under Cu-induced intracellular ROS accumulation. This supports a role of this gene in the response to oxidative stress. However, as the gene is even slightly induced under control growth conditions, we cannot rule out that it may be involved in protection against other stress conditions in AEO106. In contrast to H_2_O_2_ that passively diffuses across the cell membrane and accumulates in the exposed cells, accumulation of ROS upon exposure to Cu results from an indirect mechanism possibly involving a Fenton-like reaction, where Cu replaces iron [11, 40]. Another suggestion is that Cu disrupts iron-sulfur complexes leading to elevated iron concentrations, which then drive the Fenton reaction that is responsible for the Cu-mediated ROS accumulation [11]. Hence, it makes sense that the upregulation of the *ohr/osmC* gene was slower in the Cu exposed cells compared to the H_2_O_2_ treated cells, as it takes longer time for the cells to accumulate ROS generated by a physiological process than by diffusion.

Concomitant analysis of transcription dynamics of *tfdA* and the *ohr/osmC* gene for *C. pinatubonensis* AEO106 exposed to H_2_O_2_ and Cu, respectively, allowed us to address the question of whether this bacterium copes with stress before initiating biodegradation. Remarkably, the upregulation of the *ohr/osmC* gene in response to H_2_O_2_ coincided very accurately with a delay in expression of the catabolic *tfdA* gene (encoding the first enzyme involved in MCPA degradation). For cells exposed to Cu, the *tfdA* expression was reduced and delayed, and expression appeared in two peaks. The downregulation of *tfdA* after the first peak coincided with upregulation of the *ohr/osmC* gene. Hence both results for H_2_O_2_- and Cu exposure support that stress endurance prevails over biodegradation. Therefore, our current data for *C. pinatubonensis* strongly indicate that this bacterium cope with stress before initiating biodegradation in agreement with the notion developed by Velázquez and coworkers [19] for another important model strain for biodegradation, *Pseudomonas putida* mt-2.

A striking observation was that cells exposed to the higher concentrations of Cu (30 μM) were not able to increase their biomass as measured by OD, but they were still able to mineralize MCPA, albeit with a longer lag phase than the cells treated with 10 μM. Increased respiration without growth due to stress is a known phenomenon for bacteria exposed to hydrocarbons that induce stress by disrupting the cell membrane and thereby interfere with energy generation [41]. Cu can inactivate iron-sulfur clusters primarily of enzymes belonging to the dehydratase family [11]. These enzymes are involved in central catabolic pathways e.g. the citric acid cycle [42]. We therefore speculate that the higher doses of Cu impair energy generation, as well as regeneration of reducing powers in the form of NAD(P)H. Depletion of energy and reducing powers might in turn lead to increased activity of catabolic pathways, but at the cost of anabolic pathway activity. For *Pseudomonas* in particular, there seems to be a tight link between stress endurance and metabolic pathways that are involved in regeneration of the reducing powers from NAD(P)H [43, 44], and Obruca et al. [45] have reported a related link for *Cupriavidus necator* H16. An alternative explanation for investing in the complete mineralization of MPCA without incorporation of carbon into biomass could be that MCPA, or its degradation products, might be toxic. Hence, the removal of MCPA and degradation products by complete mineralization could be interpreted as part of the stress defense. Nevertheless, we have not been able to detect increased ROS accumulation in AEO106 in response to MCPA exposure (data not shown). In agreement with this observation, neither *Delftia acidovorans* MC1 nor *Pseudomonas putida* KT2440 experienced oxidative stress when exposed to PA herbicides in previous studies [46, 47]. However, other stress scenarios upon exposure to such chlorinated aromatic compounds than oxidative stress are also possible, as they are known for instance to impact the cell membrane and uncouple oxidative phosphorylation [47, 48].

Our current results indicate that an oxidative stress protection program is launched prior to PA herbicide mineralization in *C. pinatubonensis* AEO106. Hence, the ability of this bacterium to degrade PA herbicides under environmental conditions, where it is likely to be continuously confronted with oxidative stress inducing conditions may not primarily depend on induction of the catabolic pathways, but rather on a rapid launching of a stress response.

## Conclusion

The effect of ROS on PA herbicide degradation by *C. pintubonensis* has not been evaluated in previous studies. Here we showed that Cu, which is a relevant stress factor in many agricultural soils, leads to increased accumulation of ROS in *C. pinatubonensis* AEO106 that in turn launces a protective response against oxidative stress, including a gene homologous to *ohr* and *osmC*. The Cu-induced stress results in delayed cell growth, delayed MCPA mineralization and delayed induction of the catabolic *tfdA* gene. Hence, the data suggest that *C. pinatubonensis*, like other degraders of xenobiotic compounds, cope with environmental stress before engaging in biodegradation. The novel insight into the stress physiology of PA degrader cells adds valuable input to understanding the soil filter function and highlights the need for including agricultural management practices such as manure application in models predicting leaching of pesticides to groundwater reservoirs.

## Methods

### Strain and standard medium

*Cupriavidus pinatubonensis* AEO106 (pRO101) is a tetracyclin resistant derivative of the naturally occurring 2,4-D and MCPA-degrading soil bacterium *C. pinatubonensis* JMP134 (pJP4), which carries the genes for complete mineralization of 2,4-D and MCPA [49].

*C. pinatubonensis* AEO106 was routinely grown in Davis Minimal Medium (DMM) (Difco, USA) supplemented with 1 ml L^−1^ of trace element solution, containing 20 mg CoCl_2·_6H_2_O, 30 mg H_3_BO_3_, 10 mg ZnSO_4·_7H_2_O, 1 mg of CuCl_2·_2H_2_O, 2 mg NiCl_2·_6H_2_O, 3 mg NaMoO_4·_2H_2_O, 10 mg FeSO_4·_7H_2_O, and 2.6 mg MnSO_4·_H_2_O per liter. The medium was amended with tetracycline to a final concentration of 10 μg mL^−1^ and incubation was carried out in 500 ml flasks containing 100 ml medium at 28 °C with continuous shaking at 150 rpm.

### Growth and mineralization of MCPA under exposure to H_2_O_2_ or CuSO_4_ in pure culture

Overnight cultures of *C. pinatubonensis* AEO106 were diluted 100× in fresh DMM and incubated until an optical density at 600 nm (OD_600nm_) of approximately 0.3 was reached; then 20 ml culture was transferred to 100 ml infusion bottles. Sterile-filtered MCPA dissolved in MilliQ water (pH 7), was added to a final concentration of 25 mg L^−1^. At the same time specific stressors were added: H_2_O_2_ (final concentration 1 mM) or Cu (CuSO_4_; final concentrations 10 μM, 30 μM and 50 μM). Ring-U-^14^C labelled MCPA (specific activity 5.975 MBq mg^−1^; radiochemical purity 99.26%; IZOTOP, Budapest, Hungary) was added to a final activity of 10,000 dpm mL^−1^. A glass vial containing 1 mL 1 M NaOH (basetrap) was placed inside the bottle to trap CO_2_ produced during incubation. Upon sampling, the content of the traps was transferred to polyethylene vials and mixed with 4 mL scintillation liquid (Optiphase ‘Hisafe’3, Perkin Elmer) while fresh NaOH was added to the CO_2_ traps. Samples were analyzed in a scintillation counter (Tri-Carp 2910TR, Perkin Elmer), and total mineralization at a given time was calculated as accumulated ^14^C–CO_2_ compared to total amount of ^14^C added to the cultures.

At given time points 100 μL culture was sampled for DNA/RNA extraction (including a sampling point before addition of MCPA and H_2_O_2_ or CuSO_4_), flash frozen in liquid N_2_ and stored at −70 °C until further processing. Cell growth was measured simultaneously as OD_600nm_.

Parallel experiments were run for detection of intracellular ROS and for viability staining, but without addition of radiolabeled MCPA. All experiments were performed in triplicates and repeated twice.

### Detection of intracellular ROS and viability staining

Detection of intracellular ROS was performed using the oxidant-sensing probe, 2′,7′-dichlorodihydrofluorescein (H_2_DCF-DA; Sigma Aldrich Co.). After 30 min of incubation in the presence of either H_2_O_2_, CuSO_4_ or without any additional stressor (control), samples of 500 μL culture were incubated for 30 min at room temperature in the dark with 10 μL of 1 mg mL^−1^ H_2_DCF-DA prepared in DMSO. The samples were then analyzed by flow cytometry on a BD FACSCalibur flow cytometer (Becton Dickinson, CA) equipped with an argon-ion laser of 15 mW with excitation at 488 nm. Fluorescence from intracellular ROS-dependent oxidation of H_2_DCF-DA was recorded in the FL1 channel (515–545 nm) after gating of cells in a side scatter (SSC) vs. forward scatter (FSC) plot.

Viability staining and quantification of cells with intact or injured membranes following exposure to H_2_O_2_ or CuSO_4_ by flow cytometry was performed as in DeRoy et al. [50]. Culture samples of 500 μl were incubated in the dark for 30 min with 10 μL mL^−1^ EDTA (500 mM, pH 8) and 10 μL mL^−1^ staining solution (400 μM propidium iodide in DMSO from the BacLight Kit; Invitrogen, and 100× SYBR Green I in DMSO; Invitrogen) before flow cytometry analysis. After SSC vs. FSC gating, cells with intact membranes were distinguished from cells with injured membranes in a plot of fluorescence detected in the FL1 vs. FL3 channel (675–715 nm).

Data from at least 75,000 cells were collected from each sample and analyzed in the software CyflogicTM 1.2.1 (CyFlo Ltd.) for all flow cytometry analyses.

### Nucleic acid extraction and gene expression analyses

DNA and RNA were co-extracted using the AllPrep DNA/RNA Mini kit (Qiagen). Prior to extraction samples were treated with 10 μL of lysozyme (1 mg mL^−1^ in 10 mM Tris-Cl, pH 8) for 20 min at room temperature; otherwise the manufacturer’s protocol was followed. RNA was subsequently treated with the RQ1 DNase (Promega) followed by reverse transcription with the Omniscript Reverse Transcriptase (Qiagen) as described previously [32].

Quantitative PCR (qPCR) was performed using SYBR Green detection with the Stratagene Brilliant III Ultra-Fast SYBR® Green QRT-PCR Master Mix (Agilent Technologies) with primers targeting the *tfdA* gene (Bælum et al., 2006), and primers for the *ohr/osmC*–like gene putatively involved in an oxidative stress response in *C. pinatubonensis* AEO106 (this study). The gene was identified based on a BLAST search for ORFs homologues to genes induced under oxidative stress inducing conditions in *C. necator* H16 [15] using TBLASTX (NCBI). Annotation and protein family membership was searched for by Pfam search of translated nucelotides (http://pfam.xfam.org/).

The qPCRs were performed in 20 μL with 0.4 μM of each primer (see Table 1 for primer details) and 1 μg Bovine Serum Albumin. DNA and cDNA was diluted 1:10 prior to analysis. The PCR programs were 95 °C for 30 s, followed by 40 cycles of 95 °C for 20 s, T_a_ (Table 1) for 30 s, 72 °C for 45 s. Subsequently a melting curve was run to check for specificity of the amplification products, and qPCRs of DNase treated RNA were run in parallel to test for DNA contamination of the RNA. Standard curves were constructed based on 10-fold dilution series of DNA extracted from a culture of *C. pinatubonensis* AEO106 with a known OD, and sample *tfdA* or *ohr/osmC* gene copy numbers were calculated by relating the Ct values to these standards. No standard curves for cDNA were constructed; instead the cDNA was quantified as DNA equivalents. All standard curves had efficiencies above 80%. Gene copy numbers in cDNA samples were then converted to mRNA equivalents by accounting for dilutions in the DNase-treatment and reverse transcription steps. Finally, cell specific gene expression was calculated as mRNA copies per DNA copies.

**Table 1:** List of primers used for qPCR analyses

Target gene	Primer	Sequence (5′-3′)	Product size (bp)	T_a_ (°C)	Reference
*tfdA*	tfdA**F	GAG CAC TAC GCR CTG AAY TCC CG	260	64	[20]
	tfdA**R	CTT CGG CCA CCG GAA GGC CT			
*ohr/osmC*	B5559 F	GTC GAG CAC ATT GAC GAA GA	257	60	*This study*
	B5559 R	AGA CCG ATG TGG AGT CGT TC			

The asterisk in the tfdA primer name is merely nomenclature, and does not reflect any reference to statistical or other measures

## Abbreviations

Cu: Copper
H_2_DCF-DA: 2′,7′-dichlorodihydrofluorescein diacetate
MCPA: 2-methyl-4-chlorophenoxyacetic acid
OD: Optical density
PA: Phenoxy acid
ROS: Reactive oxygen species
